# Can Data Mining Improve Methane Correction Factors for Urban, Nonsewered Sanitation?

**DOI:** 10.1021/acs.est.6c03733

**Published:** 2026-07-02

**Authors:** Michael Vogel, Linda Strande

**Affiliations:** 28499 Eawag: Swiss Federal Institute of Aquatic Science and Technology, Sandec: Department of Sanitation, Water and Solid Waste for Development Überlandstrasse 133, Dübendorf 8600, Switzerland

**Keywords:** anaerobic degradation, greenhouse gas, emissions, fecal sludge management, intergovernmental panel on climate change, storage, treatment, wastewater

## Abstract

Nearly half of the world’s population relies on nonsewered sanitation, and in urban areas, intermittent onsite storage of wastewater prior to road-based transport to treatment is common. Anerobic biological activity during storage generates methane, contributing to greenhouse gas emissions and posing challenges for climate mitigation. Current estimates of methane emissions rely on methane correction factors (MCFs) in the Intergovernmental Panel on Climate Change (IPCC) guidelines that are based on theoretical assumptions and increasingly viewed as not valid. In this Perspective, we use 1349 data points from onsite storage of wastewater in 11 cities to show that obtaining accurate city-wide estimates is complicated by highly variable properties of in situ wastewater and storage conditions and hence biological production of methane. When it comes to greenhouse gas emissions from nonsewered sanitation, the sector has so far focused on storage, but as evidence suggests these estimates are too high, we propose that emphasis should in fact be placed on the entire service chain.

## Graphical abstract

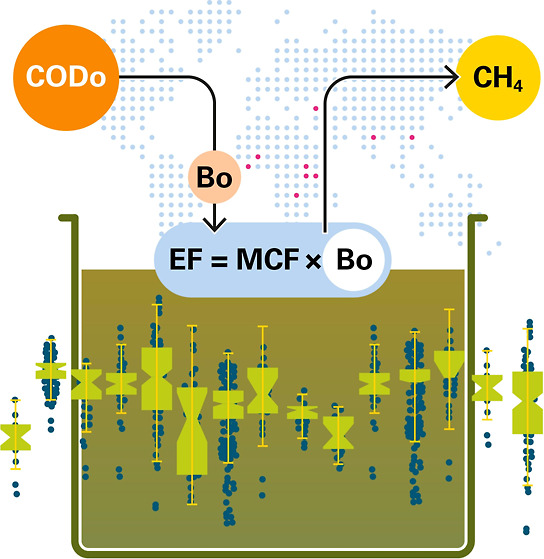

## Introduction

Globally, greenhouse gases (GHGs) that leak from municipal wastewater service chains represent a significant source of carbon emissions, and to reach net-zero targets, we must pinpoint where and why these emissions occur.^1^ Almost half of the world’s population now relies on nonsewered sanitation, including 60–90% of municipal wastewater in urban areas of lower- and middle-income countries.^2^ In urban areas, nonsewered wastewater is intermittently stored onsite in containments (tanks and pits) until they become full and is then transported by road to treatment.^3^ Attempts to estimate emissions typically rely on intergovernmental panel on climate change (IPCC) guidelines, but the assumptions that these calculations are based on are increasingly scrutinized. Estimates are focused on methane (CH_4_) that is generated from the anaerobic degradation of organic matter in wastewater during storage,^4^ as nitrous oxide (N_2_O) has not been detected in field studies,^5–7^ and carbon dioxide (CO_2_) is considered as biogenic carbon.^8^ However, we currently lack sufficient knowledge of anaerobic biological processes in nonsewered service chains to quantify emissions.^9^ Rough calculations have recently led to wide-ranging estimates, such as 0.06 to 110 g of CH_4_ per capita-day from onsite storage of wastewater,^10^ and nonsewered sanitation contributing globally to 0.3%–12.5% of anthropogenic CH_4_ emissions.^11^ It is not clear whether sewer-based transport to treatment with centralized treatment, or onsite storage with road-based transport to treatment, has higher or lower GHG emissions, as both have been reported over the range of 50–185 kg CO_2_ equivalents per capita-day.^12–14^ Reasonably accurate estimates are needed for practitioners and policy makers to make evidence-based decisions for the mitigation of GHGs from sanitation, to evaluate where in the service chain to target mitigation efforts, and to determine which technologies, scales, and management models will truly result in reductions of emissions—such as simplified sewers with decentralized treatment,^15^ container-based sanitation,^16^ or nature based solutions.^17^ As is the trend with centralized, sewer-based systems, climate finance mechanisms like the voluntary carbon market could channel private sector capital to credits for reduced emissions and resource recovery from gas capture,^18^ while simultaneously increasing access to safely managed sanitation. Overall, research efforts to uncover the contribution of nonsewered sanitation to CH_4_ emissions have remained unsuccessful,^5,7,19–22^ increasing the risks of “Garbage In, Garbage Out” models and impeding efforts to unlock climate funding to improve sanitation.

In this article, we compile published data to highlight inaccuracies in methane correction factors (MCFs) in IPCC guidelines. Using 1349 data points from 11 countries, we examine the relations between demographic and technical survey data and wastewater properties, discuss whether this new approach could improve the accuracy of MCFs, and identify how changes can be rapidly implemented to improve sampling strategies and city-wide estimations of CH_4_ emissions.

### Current Framing of IPCC Guidelines

In rural areas, pit latrines and septic tanks are based on established designs with adequate land available for treatment.^3^ In contrast, in urban areas where land is scarce, pits and tanks are intended for storage, they lack consistency in their design, and construction is highly variable and poorly documented.^23^ Biological degradation processes during storage are not well understood, but tend to plateau during storage, and are not as complete as previously assumed.^24^ Nonsewered wastewater going into pits and tanks is dilute (<5% total solids (TS)) and includes cooking, bathing, and cleaning water (gray water).^25^ It is therefore highly variable, with variations in properties and accumulated volumes far greater (1–2 orders of magnitude) than municipal wastewater that is partially homogenized during transport in sewers.^26,27^ Normal distributions are not applicable, simple averages are not valid, and estimates cannot be projected directly by population, greatly complicating attempts to make reasonably accurate projections at city-level.^28–30^ Yet the most recent IPCC guidelines^31^ attempt to capture this variability with only four categories of methane correction factors (MCFs) for estimating CH_4_ emissions from wastewater during storage, with no distinction for urban or rural locations, with or without onsite, land-based treatment. This includes one category for “septic tanks”, and three categories for “pit latrines” based on dry or wet climate, high or low groundwater, dry or flush toilet, and “few or many” users.^31^

Variables defined in the IPCC guidelines include the chemical oxygen demand (COD_o_) of wastewater entering storage, the maximum amount of COD_o_ that could be stoichiometrically converted to CH_4_ (B_o_), and the MCF. The MCF adjusts for the actual CH_4_ produced from anaerobic degradation based on the in situ wastewater and storage conditions controlling biological processes.^31^ The emissions per COD_o_ loading are therefore calculated as

$$EF_{j}=B_{O}\times MCF_{j}$$

where

$$EF_{j}=emission\;factor\left(\frac{kgCH_{4}}{{kgCOD}_{O}}\right)$$

j=eachtreatmentordischargepathway,system,orcontainment

$$B_{O}=maximum\;CH_{4}\;producing\;capacity\left(\frac{kgCH_{4}}{{kgCOD}_{O}}\right)$$

$$MCF_{j}=methane\;correction\;factor\;\left(fraction\right)$$

Measuring in situ wastewater properties and quantifying emissions from tanks or pits across an entire city are constrained by logistics, time, and budget.^1^ Therefore, MCF values in the IPCC guidelines are based on expert judgment, with limited to no field data.^7,31,32^ Efforts to improve MCFs include measuring CH_4_ emissions from individual storage containments in the field.^5–7,20,32^ However, given the high variability of in situ wastewater and storage conditions, scaling up to city-wide estimates from point source emissions cannot result in reasonably accurate estimates unless MCF values are directly coupled with the actual conditions that are controlling anaerobic biological processes.

### Root of Methane Correction Factors Inaccuracy

The *B*_o_ (maximum CH_4_ producing capacity value) of 0.25 gCH_4_/gCOD is generated from the stoichiometric calculation of the complete conversion of glucose (C_6_H_12_O_6_) to CH_4_. In comparison, biomethane potential (BMP) evaluates, through standard laboratory methodology, the maximum amount of COD in wastewater that can be converted to CH_4_ (Supporting Information S1), designated here as *B*_0,meas_ (Figure 1). BMP tests are conducted under ideal conditions for CH_4_ production through anaerobic degradation (35–37 °C, constant mixing, anaerobic digester sludge as an inoculum).^33^ *B*_o,meas_ values reported for feces and urine mixtures are only 0.082–0.132 gCH_4_/gCOD (Table 1), and with lower temperatures (20–37 °C), and wastewater from storage in pits or tanks as inoculum, they even decline to 0.009–0.116 gCH_4_/gCOD (Table 1), illustrating how the stoichiometric method can overestimate COD quantities converted to CH_4_ in wastewater.

**Figure 1:**
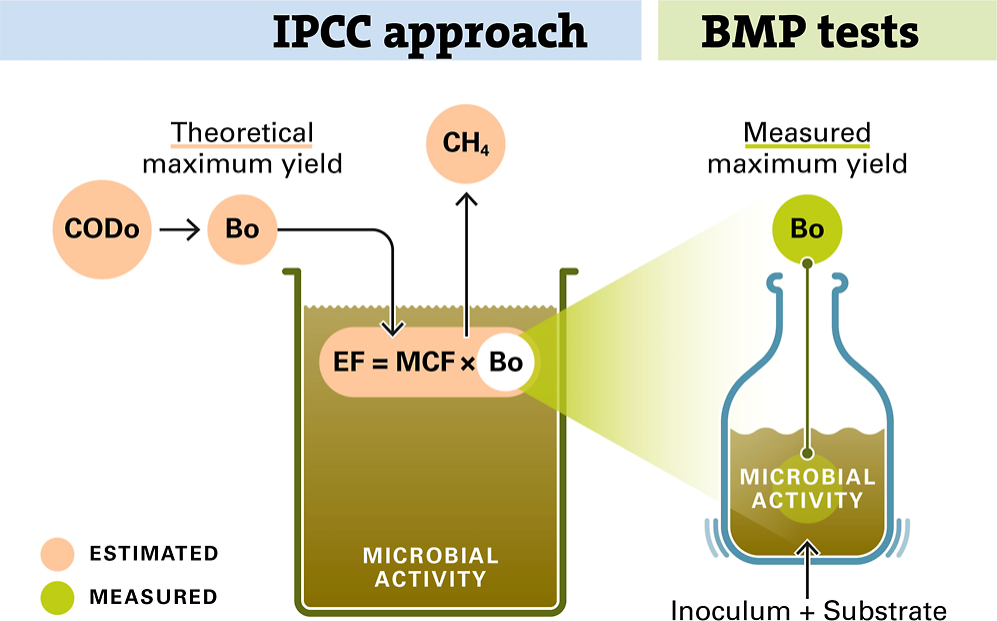
Conceptual model for estimating CH_4_ emissions from wastewater during storage in containments in the IPCC guidelines,^31^ and relation of *B*_o_ and *B*_0,meas_ from biomethane potential (BMP) tests that measure maximum methane emissions under controlled conditions.

**Table 1:** Reported and Calculated Values from Biomethane Potential (BMP) and Modified BMP Tests of Feces and Urine^k^ ^l^

Substrate	Inoculum	Temp	B_0,meas_ gCH_4_/gCOD	B_o_ gCH_4_/gCOD	MCF_meas_	MCF
Biomethane potential (BMP) tests						
Feces + urine	AD sludge	37 °C	0.132^a^	0.25^f^	-	-
Feces	AD sludge	35 °C	0.082^b^	0.25^f^	-	-
Feces + saw dust	AD sludge	35 °C	0.110^c^	0.25^f^	-	-
Modified biomethane potential (BMP) tests						
Feces + urine	Tank WW	20 °C	0.116^d^	0.25^f^	0.46^d^	0.5^g^ (0.4–0.72)
Feces + urine	Pit WW	20 °C	0.009^d^	0.25^f^	0.04^d^	0.7^h^ (0.7–1.0)
Feces + urine	Pit WW	37 °C	0.081	0.25^f^	0.32^d^	0.5^i^ (0.4–0.6)
Feces	Tap water	25–35 °C	0.015^e^	0.25^f^	0.06^e^	0.1^j^ (0.05–0.15)

a Maqbool et al. (2024)^46^

b Kim et al. (2019)^47^

c Riungu et al. (2019)^48^

d Sam et al. (2022)^42^

e Singh et al. (2021)^41^

f Bo Values from IPCC (2019).

g MCF for Septic Tanks from IPCC (2019).

h MCF for Pit latrine, wet climate/flush toilet, and high groundwater from IPCC (2019).

i MCF for Pit latrine, dry climate low water table, many users from (IPCC 2019).

j MCF for Pit latrine dry climate, low water table, 3–5 users from IPCC (2019).

k B_0,meas_ is the CH_4_ production from BMP tests, B_O_ is the stoichiometric maximum CH_4_ yield in the IPCC guidelines, MCF_meas_ is the calculated difference of actual CH_4_ production versus B_o_, and MCF are from the IPCC guidelines.

l AD = anaerobic digestor from centralized, sewer-based wastewater, WW = wastewater during storage in containment from nonsewered sanitation.

The MCF is a dimensionless value in the IPCC guidelines, intended to correct *B*_0_ from the stoichiometric value to the actual quantity of converted COD (Table 1). However, using the IPCC values can lead to predicting CH_4_ emissions that are as high as the *B*_0,meas_ values, as MCFs can be equal to one (Table 1). In reality, this cannot occur due to the complexity and bioavailability of organic matter in wastewater, including lignin, polysaccharides (cellulose and hemicellulose), protein, lipids, extracellular polymeric substances, and microbial cells.^34–36^ To correct this, a recent desk-based study added a blanket “percentage reduction” term of 0.7 to all MCFs, applying the idea that 70% of COD is degraded in pits.^14^ However, this was solely based on results with dry pit latrines with an aerobic surface layer in one rural area of South Africa,^37,38^ and other desk-based estimations have led to even higher emissions than the IPCC guidelines.^39^ Conversely, laboratory studies have reported a lower range of 30–62% degradation.^40–42^

For a more nuanced view, we compiled reported MCF_meas_ values from modified BMP tests (Table 1), comparing the amount of CH_4_ produced to the stoichiometric values (MCF_meas_ = *B*_o,meas_/*B*_o_). The MCF_meas_ values are likely an overestimation of MCFs as they are the maximum CH_4_ produced under laboratory conditions with mixing. The MCF_meas_ values ranged from those of IPCC guideline MCFs to an order of magnitude lower than these. Developing evidence-based MCFs now requires a finer understanding of in situ conditions that could inhibit the anaerobic degradation of organic matter during storage, including a lack of mixing, fluctuations in pH, limitations in macro- or micronutrients, redox (reduction–oxidation) conditions,^43,44^ and the presence and function of microbial communities.^45^

### Global Properties of In Situ Wastewater Driving CH_4_ Emissions

While the variability of stored wastewater properties is widely accepted, we still lack information about patterns sustaining such variations. To provide a synthesized view of this matter, we analyzed the variability of wastewater properties in pits and tanks within and between cities, using data of TS, pH, ammonium (NH_4_^+^), volatile solids (VS), COD, and electrical conductivity (EC), collected over the past decade by our group, from 1349 different storage containments across 11 countries (Figure 2). The data are from nine separate studies conducted during this time that collected the same questionnaire data and laboratory characterization of composite samples of wastewater from pits and tanks. This is the largest data set of its type that we are aware of, and for data mining, we applied statistical synthesis, association analysis, and clustering, to discover patterns and relationships in the data (the methodological workflow is presented in detail in Supporting Information S2). We specifically explored how properties of wastewater and storage conditions might influence anaerobic biological degradation and resulting MCFs, by focusing on TS, pH, and NH_4_^+^, and their role in anaerobic degradation (Figure 2A–C). As COD, VS, and EC were strongly correlated to TS and NH_4_^+^ (Spearman correlation matrix in Supporting Information S4), they did not provide additional information and were excluded from further analysis.

**Figure 2:**
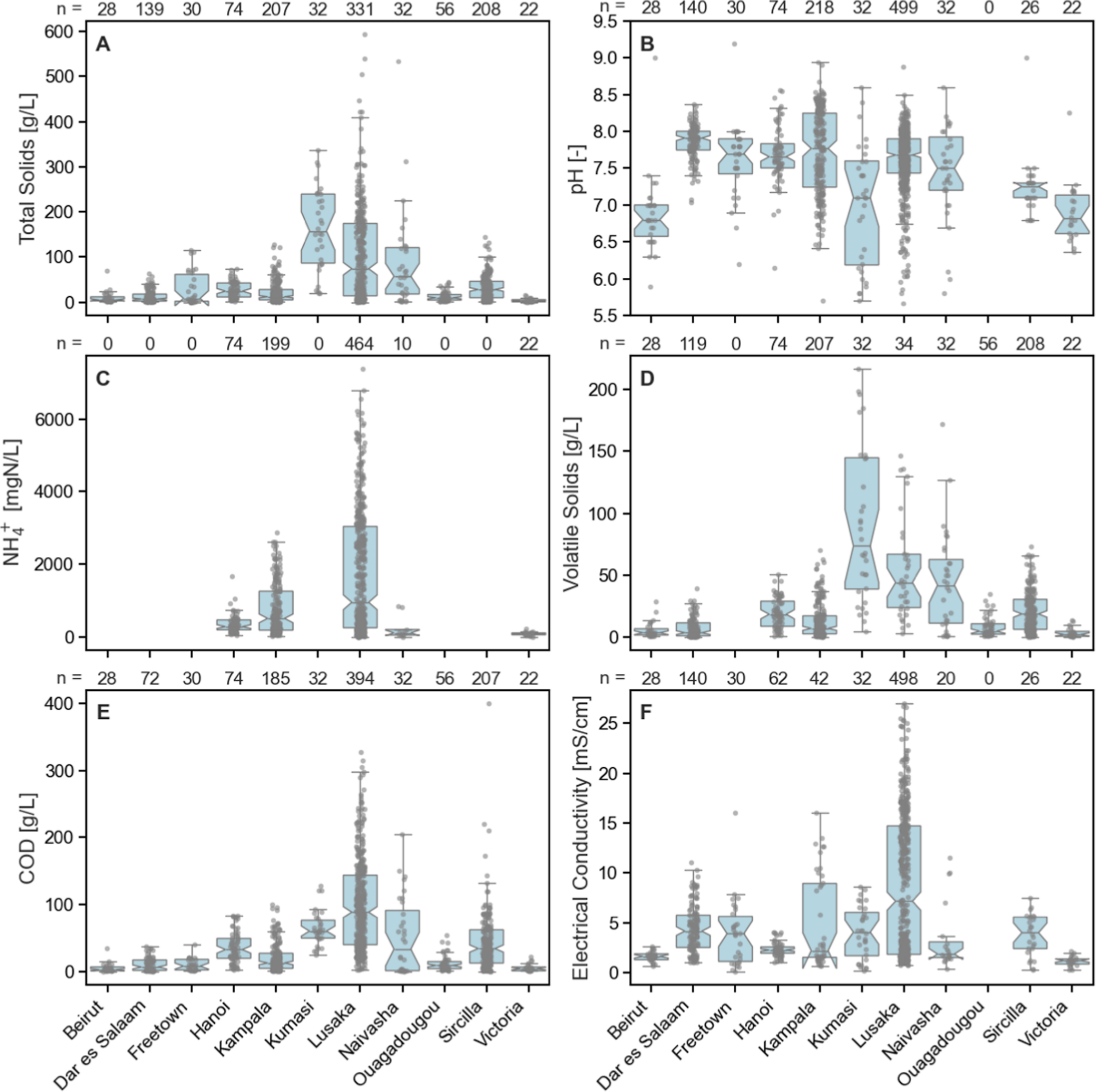
Overview of physical and chemical parameters of wastewater stored in onsite containments (pits and tanks) in urban areas of 11 different countries.

TS influences CH_4_ production, with reduced anaerobic degradation at high TS due to the limited diffusion of volatile fatty acids. Generally, in process-controlled anaerobic digesters with mixing, higher CH_4_ production is reported at 20–120 gTS/L, with a peak at 80 gTS/L,^49,50^ and decreasing production when TS increases from 100 to 250 gTS/L and beyond.^51^ Within the entire data set, 80% of the wastewater was very dilute with a TS lower than 5% (50 gTS/L), with only 10% higher than 135 gTS/L and 3% higher than 250 gTS/L.^52^ Interestingly, a positive correlation was previously observed at lower concentrations of TS in tanks in Hanoi, Vietnam, between total suspended solids (TSS) and CH_4_ emissions with 5–35 gTSS/L.^20^

Anaerobic degradation, and therefore CH_4_ production, can be inhibited below pH 6.5,^53^ and at higher pH, it can be inhibited by free ammonia nitrogen (FAN, NH_3_).^53^ Values for pH ranged from 5.5 to 9 across the 11 cities, with a median of 7.7, and only 10% of data points below 6.5. For comparison, in anaerobic reactors, a drop in pH from 7 to 5.5 can reduce methanogenic activity by 72%.^54^ We calculated FAN using the in situ pH and NH_4_^+^, and an assumed temperature of 24 °C (Supporting Information S5), which resulted in in situ FAN above 68 mgN/L in 20% of containments, with a high of 500 mgN/L. In anaerobic reactors, a FAN of 60 mgN/L can inhibit methanogenesis by up to 75%.^55,56^ However, methanogens can also adapt to FAN, with reports of inhibition above 1100 mg/L FAN.^57^

Although the exact roles of TS, pH, and NH_4_^+^ on CH_4_ production are not yet known, the range of reported values illustrates the importance of examining in situ properties of wastewater for grouping MCFs, rather than only technical and demographic data such as dry or wet climate, high or low groundwater, dry or flush toilet, and few or many users.

### Relationships between Categories and TS, pH, and NH_4_^+^

In the IPCC guidelines, technical and demographic data are useful to group MCFs as this type of information is relatively inexpensive to collect via surveys. If linked statistically with wastewater properties, it could provide a reliable way to scale up estimates to the city level. This was demonstrated in Kampala, Uganda, where household income level, water connection, and gray water going into containments are statistical predictors of TS.^58^ Building on this, we evaluated whether other technical and demographic survey data across the 11 cities were statistically related to TS, pH, and NH_4_^+^ (Supporting Information S3), and almost all categories did show significant relationships (Table 2 and Supporting Information S6–S8). We further mined the largest city data sets for Kampala and Lusaka. Although the patterns of statistical significance for demographic and technical categories differed from those of the complete data set (Table 2), this demonstrates that similarities between cities and regions do exist.

**Table 2:** Summary of Results of Welch’s ANOVA between Survey Data and TS, pH, and NH_4_^+^ ^a^

		All containments			Pits			Tanks		
	Survey data	All cities	Kampala	Lusaka	All cities	Kampala	Lusaka	All cities	Kampala	Lusaka
TS	Containment type	***	**	***						
	Fully lined	*-	*-	NA	*-	NA	NA			
	Overflow	***	**	***	*	NA	*-	*-		
	Water connection	***	-	***	-	NA	-	-	-	-
	Gray water added	**	**	**	-	**	**	-	-	-
	Flush type	***	-	***	***	NA	-	***	-	-
	Income level	-	**	**	**	***	-	*-	-	-
	Building usage	***	***	***	***	*-	***	-	**	
	Users >10	*-	-	-	**	-	-	**	-	-
pH	Containment type	***	***	-						
	Fully lined	-	-	-	*	NA	-	*	NA	NA
	Overflow	*	**	-	-	NA	-	***	-	-
	Water connection	-	***	-	-	NA	-	***	**	***
	Gray water added	***	***	-	-	-	-	-	-	-
	Flush type	***	**	*-	NA	-	***	-	*	
	Income level	***	***	-	***	*-	***	-	-	
	Building usage	*	**	-	-	-	-	*	*	*
	Users >10	**	-	-	***	-	-	-	-	-
NH_4_^+^	Containment type	***	***	***						
	Fully lined	**	NA	**--**	NA	-	NA	NA	NA	
	Overflow	***	NA	***	-	NA	-	-	NA	-
	Water connection	***	NA	***	-	NA	-	-	NA	-
	Gray water added	***	***	***	-	-	**	*	*-	
	Flush type	***	NA	***	-	NA	-	-	NA	*
	Income level	***	***	***	**	-	-	-	**	-
	Building usage	***	***	***	**	-	-	***	***	***
	Users >10	**	*-	-	-	**	-	***	-	**

a Symbols indicate the level of significance: * for *p*-value <0.05, ** for *p*-value <0.01, *** for *p*-value <0.001, - for *p*-value >0.05 (no statistical significance), and NA not available (sample size *n* < 30). Categorical values for survey data were containment type = pit or tank; fully lined, overflow, water connection, and gray water added = yes or no; flush type = cistern flush, pour flush or dry toilet; income level = very low, low, middle or high; building usage = household, schools, public toilet or commercial; and users = <10 or ≥10 (Supporting Information S3).

Based on MCF categories of “pits” and “tanks” in the IPCC guidelines, we further analyzed the data set according to these across all cities, or for Kampala and Lusaka separately (Table 2). With all cities, in situ TS, pH and NH_4_^+^ wastewater characteristics in pits and tanks had significant associations between toilet flush type (defined here as cistern, pour-flush, or dry toilet), building usage (household, schools, public toilets, or commercial—e.g., factory, office, mall, restaurant, and house of worship), income level, and numbers of users, illustrating their potential usefulness in defining finer MCF categories. In addition to toilet flush, technical survey data reflecting different levels of water usage, such as direct water connection at building, and whether containments also hold gray water, could further refine such categories. The same relations were not found within individual cities, potentially due to limited sampling sizes.

Notably, the current IPCC guidelines only consider household pits and tanks, when approximately 50% of municipal wastewater typically comes from nonhousehold sources.^59^ Storage of wastewater in pits and tanks from these unaccounted urban sources should also be included for more accurate city-wide estimates of GHG emissions. The relation of building usage to wastewater properties indicates the potential for different emission profiles that could be useful in defining the MCF categories.

Evidently, categorizing only by pit or tank does not capture the variability of physicochemical properties of wastewater. Although TS, pH, and NH_4_^+^ relate to demographic and technical data, their exact relation to MCFs in the IPCC guidelines is not clear. Certainly, categories of MCFs for pits and tanks merit further refinement to capture the variability, as what is commonly referred to as “septic tank” actually refers to several types of containment from fully sealed tanks with no outflow, to cesspits with permeable surfaces.^3^ Potentially, rather than further dividing categories of containment types (e.g., fully sealed, unlined, and overflow), an approach that first evaluates the in situ properties of wastewater controlling CH_4_ production, and then identifies whether technical and demographic data are predictors, could improve the accuracy of MCFs.

### Defining Categories by Clustering of In Situ Properties

Next, we assessed statistical clustering of TS, pH, and NH_4_^+^ that could serve to define broader, but more refined, categories of MCFs (Supporting Information S3 and S9). Because wastewater properties were highly variable in Lusaka, clustering was driven by city, and we focused individually on Kampala and Lusaka. We identified three clusters in Kampala (Figure 3A–C): Cluster 1 with low TS, range of pH, and low NH_4_^+^ (median values of 6 gTS/L, 7.4 pH, and 221 mgN/L); Cluster 2 with high TS, range of pH, and low NH_4_^+^ (64 gTS/L, 7.1 pH, and 333 mgN/L); and Cluster 3 with medium TS, high pH, and high NH_4_^+^ (24 gTS/L, 8.4 pH, and 1587 mgN/L). Potentially, the higher median TS concentration in Cluster 2 is still low enough to not be limited by mass transfer (64 gTS/L), whereas the higher median calculated FAN of 185 mgN/L in Cluster 3 could restrain CH_4_ production in comparison to only 2 mgN/L each for clusters 1 and 2, although it is within the range reported for adapted resilience.^57^ The difference in TS with similar NH_4_^+^ in Clusters 1 and 2 was not expected and could indicate other useful information for clustering MCFs, such as which toilets people use more for defecation than urination. The high FAN in Cluster 3 could also indicate these toilets are used predominantly for urination. Juxtaposing COD, sCOD, and FAN (not included for cluster definition) illustrates that the relative distribution ranges are similar (Figure 3D–F), with a Spearman correlation of 0.77 between COD and TS (Supporting Information S4).

**Figure 3:**
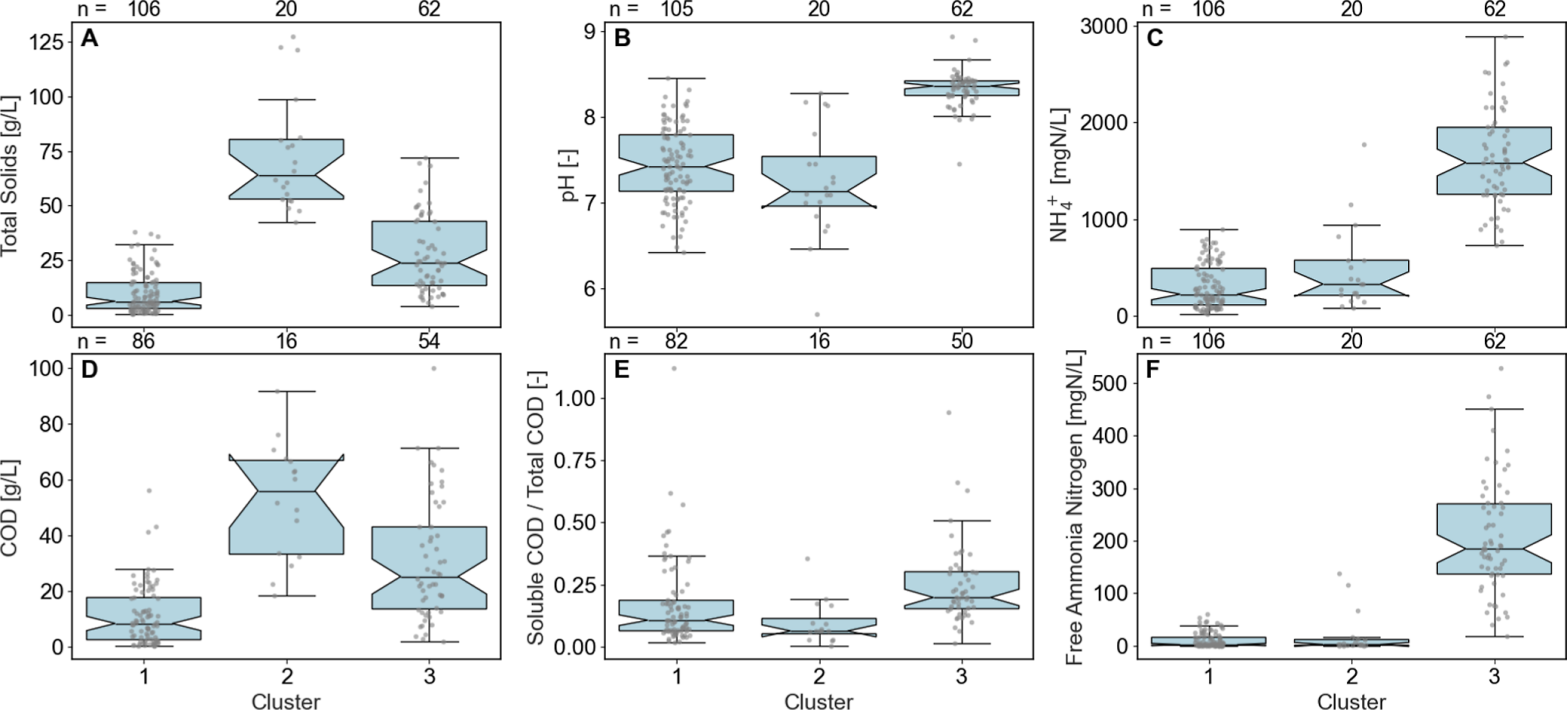
Notched box plots illustrating three clusters of physicochemical properties of wastewater, based on total solids (TS), pH, and NH_4_^+^ (A–C). COD, sCOD, and calculated FAN (D,E) included for illustration.

We then assessed whether the three clusters were related to technical and demographic information (Supporting Information S10) that could help to predict these clusters. For Kampala Clusters 1 and 2, storage was mostly in tanks (85% and 65% of data points, respectively), with gray water addition in containments (65% and 55%, respectively), and toilets had a cistern flush (67% for both). Wastewater was mostly from households in clusters 2 and 3 (60% and 77%), contrary to cluster 1 (47%). With cluster 3, storage was mostly in pits (84%) and without gray water (79%). In this cluster, households appeared to have reduced access to water and were mainly very low- or low-income, whereas in clusters 1 and 2, households were also distributed among middle- and higher-income. The higher fraction of household sources in cluster 3 could explain the differences in COD (defecation) and NH_4_^+^ (urination) with other clusters, reflecting the more prevalent defecation at home than outside.^60^

Applying the same methodology to Lusaka (Supporting Information S11–S14) resulted in Cluster 1 having low TS, range of pH, and low NH_4_^+^ (19 gTS/L, 7.7 pH, and 250 mgN/L), with 75% of wastewater from households, 85% reported as tanks, and 78% of toilets had a cistern flush. Cluster 1 in Lusaka and Kampala were the only clusters that had overlapping values of TS, pH, and NH_4_^+^. They also both exhibited the highest fraction of nonhousehold buildings, tanks, and indicators of higher water usage. Cluster 2 in Lusaka had high TS, low pH, and high NH_4_^+^ (162 gTS/L, 6.4 pH, and 2172 mgN/L); wastewater was mostly from households (87%), in pits (78%), with added gray water into containments (64%), and only 18% of toilets had a cistern flush (82% dry toilets). Cluster 2 in Lusaka had much higher TS and NH_4_^+^ than in Kampala, but due to the difference in pH, both locations showed low values of calculated FAN (2 and 3 mgN/L). Lusaka displayed a much higher fraction of pits than tanks and dry toilets. However, both cities had similar fractions of added gray water, illustrating that the categories “pit” and “flush” are not reflective of total water inputs. Lusaka’s Cluster 3 had high TS, pH, and NH_4_^+^ (192 gTS/L, 7.8 pH, and 3229 mgN/L), with 95% of wastewater from households, 91% reported as pits, 34% had addition of gray water, and 88% of toilets were dry. This was similar to Cluster 3 in Kampala (highest proportion of pits, lowest of gray water addition). Although the median NH_4_^+^ in Cluster 3 was double in Lusaka (3230 mgN/L) than in Kampala (1590 mgN/L), the calculated FAN was higher in Kampala (185 mgN/L vs 93 mgN/L), due to differences in pH.

Overall, there were general differences between Kampala and Lusaka. In Kampala, there was a much higher fraction of very low-income households, number of users per toilet, and commercial and public toilets; the wastewater was much more dilute (TS, Figure 2), and pour flush generally dominated, but the gray water entering containments was similar for both cities and all three clusters. Household income level was influential in Kampala but not Lusaka. The data from Kampala and Lusaka were from two separate studies and were not set up for comparison. In addition, although demographic and technical survey data are clearly predictors of TS, pH, and NH_4_^+^ individually (Table 2), and additional indicators such as flush type, gray water addition, and building usage provide useful linkages to in situ characteristics, the relations are not as strong when clustering the data by in situ properties (Supporting Information S10 and S13).

Based on these findings, it is unclear if similarities indicate whether MCFs can be globally or regionally developed or should rather be based on the actual properties of wastewater in each individual city. Evidently though, our clustering analysis revealed important trends for CH_4_ emissions that are currently not captured by MCFs in the IPCC guidelines but appear useful to inform sampling strategies. In the future, this approach could be developed for global default values, with refinement at the city or regional level, based on readily obtainable information on demographics, water usage, and sanitation typologies. Paired with laboratory characterization, relationships between varying wastewater properties due to management and usage practices can be compiled. In an iterative fashion, the increased availability of data will increase the accuracy and scalability of the approach.

## Implications to Improve the Categories of Methane Correction Factors

The IPCC guidelines provide a solid starting place for estimating city-wide CH_4_ emissions from sanitation; however, they are not based on the most up-to-date scientific evidence,^24^ including misconceptions that storage in pits and tanks in urban areas is analogous to pit latrines and septic tanks associated with land-based treatment.^3^ For the onsite storage of nonsewered wastewater in urban areas, the guidelines could be greatly strengthened with categories of MCFs based on the actual in situ properties of wastewater. Consequently, we propose immediate actions for the refinement of MCFs in ongoing assessment cycles of the IPCC guidelines, that together with targeted sampling strategies, and consideration of the entire service chain, could more rapidly move the sector forward, toward reasonably accurate estimates of GHG emissions. Specifically, we recommend implementing the following:

- Create distinct categories for urban and rural areas, as the current approach using demographic and technical data to predict MCFs does not account for the large variability of in situ storage conditions in cities, where the onsite storage of wastewater is highly variable and poorly documented.
- Include wastewater from nonhousehold sources (e.g., schools, commercial, and public toilets) in city-wide estimates of GHG emissions, as current methods fail to account for these, when their usage patterns and in situ properties differ from those of household wastewater.
- Survey data that are linked to water usage should be included, as wastewater within the same demographic and technical groupings are still highly variable. In addition to flush or dry toilets, pour-flush versus cistern flush, connection to drinking water, and graywater addition to containments are important predictors of in situ properties of wastewater in storage.
- Consider that storage in pits and tanks is not equivalent to capture, as containment linings are often permeable and overflows can enter the environment, including via illegal drainage.^20^
- Refine MCF values to reflect that storage is not a treatment. Biological degradation during storage is less than previously thought, plateaus after 1 week,^42,61^ and with continual fresh inputs of wastewater, the time since last emptied is also not a predictor of stabilization.^24^ Data collected on emptying intervals should reflect these timelines.
- Avoid transferring concepts from planning of sewer-based wastewater management directly to nonsewered sanitation. When reporting per capita GHG estimates, the common assumption that one household resident equals one population equivalent loading of COD for an individual toilet is not valid, as people move around, using multiple toilets per day.^60^ Because of highly variable user numbers, per capita estimates cannot be estimated on a per toilet or containment basis. To account for this variability and movement within a city, potentially GHG emissions from storage of wastewater could be estimated for entire cities based on modified MCF groupings and then normalized on a regional basis to population equivalents.

In this Perspective, we used an innovative approach to examine in situ wastewater during storage, focusing on the most widely available data type, namely levels of TS, pH, and NH_4_^+^. Targeted sampling strategies need to increase the depth of data collection, rather than the breadth, to improve our mechanistic understanding of anaerobic degradation during storage, concentrating on methods that are directly comparable between cities. These results can then be used to form clusters or categories of MCFs based on the actual levels of anaerobic biological degradation that are producing CH_4_. This includes

- An increase in the depth of data collection to include anaerobic conditions (e.g., dissolved oxygen and redox potential), bioavailability of organic matter (e.g., BMP, biochemical oxygen demand, and dehydrogenase activity),^46,62^ inhibitors of anaerobic digestion (e.g., sulfate/sulfide), and present and active microbial communities^20,45^ will provide ways to account for the high variability when scaling emissions to city or regional levels.
- Parameters that are readily quantified at lower costs should be included. Survey and sensor data could increase practicality for future sampling campaigns and scalability of results.
- Quantify organic matter going into containments, not just in situ values. On the basis of the IPCC assumption of a steady-state model, the difference between influent and in situ values is the amount that is anaerobically biologically converted to CH_4_. Point source measurements of CH_4_ emissions need to validate quantified emissions with influent values of organic matter, together with what was reported in controlled laboratory settings, and evaluate whether reported emissions are realistic based on possible levels of anaerobic degradation.
- Utilize analytical methods to truly understand degradation mechanisms and the role of inhibition during storage, as BMP tests confirm that MCFs are not reflective of the limited degradation and subsequent emissions during storage. Molecular-based analytical methods that allow more direct examination of biological populations and mechanisms within environmental samples are rapidly evolving while simultaneously decreasing in cost. Metagenomics and stable isotope labeling could be used in situ, and metatranscriptomics and qPCR in controlled laboratory settings, for improved understanding of metabolic pathways.
- Include dissolved CH_4_, not only emissions. Measuring emissions with flow chambers do not capture the fractions of CH_4_ that are dissolved or trapped in aggregates and then released, for example, during emptying.^63^ Evaluate at what level nonbiogenic sources of organic matter, such as soaps or detergents, are contributing to emissions.^8,64^

Given that CH_4_ production during storage appears to be overestimated, we advocate for the need to widen the focus on the entire service chain. This would account for incomplete storage, whereby some organic matter in wastewater enters the environment, inadvertently or illegally. Simultaneously, fractions of dissolved CH_4_ that are largely unquantified are also likely released to the atmosphere during emptying with vacuum trucks, delivery at treatment, and settling-thickening tanks, based on previous studies.^65^ Additionally, the biodegradable organic matter and nitrogen forms remaining undegraded in wastewater during storage are likely released as GHGs from biological degradation in downstream treatment processes. Overall, CH_4_ emissions resulting from passive anaerobic treatment (e.g., settling-thickening tanks and stabilization ponds)^9^ remain largely unquantified for nonsewered sanitation. Accurately estimating CH_4_ emissions along the entire service chain would provide better evidence to unlock development funding focused on reducing GHG emissions.

Accurate citywide estimates of GHG emissions from urban sanitation in lower- and middle-income countries are further exacerbated in informal settlements, which are often located in low-lying areas that are prone to flooding. If excreta are flushed or illegally drained directly into the environment, it is possible that CO_2_ equivalent emissions could actually decrease due to passive aerobic degradation producing mainly biogenic CO_2_. The climate discussion in these areas should rather be around adaptation to robust solutions that remain functional and hygienic during extreme weather events and potential credits for gas capture and resource recovery.

The data is not yet there for practitioners and policy makers to make evidence-based decisions for the mitigation of GHGs from sanitation, and unlock access to carbon credits and other climate funding. It is not clear what baseline emissions from sanitation cannot be eliminated, and whether sewer-based transport to treatment with centralized treatment, and onsite storage with road-based transport to treatment, have higher or lower GHG emissions.^12–14^ This evidence is needed to evaluate solutions for mitigation, such as more at-source decentralized treatment of wastewater, simplified sewers, container-based sanitation, nature-based solutions, separate management of gray water, or in situ biological CH_4_ mitigation. Without this, we run the risk of misguided mitigation attempts, that do not result in reduced emissions. Additionally, we need to ensure that climate considerations are synergistic with the overall protection of public health, with provision of basic rights to sanitation as a first step,^66^ and equitable distribution of responsibilities, for example, acknowledging that the entire continent of Africa accounts for only 3% of global GHG emissions.

## Supplementary Material



## Notes

Overview of the literature that was used for BMP analysis, including properties of inoculum; overview of the literature and data set where the characteristics of the 1349 different storage containments across 11 countries were compiled from; details of the methodological workflow that was used for statistical synthesis, association analysis, and clustering to identify patterns and relationships in the data; Spearman correlation matrix of physicochemical parameters; calculated free ammonia nitrogen (FAN); and summary of Welch’s ANOVA, Silhouette plots, grouped information from questionnaires, and notched box plots of clusters (PDF)
