# Supplementary material for: Can Data Mining Improve Methane Correction Factors for Urban, Nonsewered Sanitation?
Source: Environ Sci Technol. 2026 Jul 2;60(27):19041–50. doi: 10.1021/acs.est.6c03733 (PMC13374094; doi:10.1021/acs.est.6c03733)
Supplement: Supplementary file 1 [file es6c03733_si_001.pdf]

Supplementary information

Can data mining improve methane correction factors (MCFs) for urban, non-sewered sanitation?

Michael Vogel[1]
Linda Strande[1,*]

[1]Eawag: Swiss Federal Institute of Aquatic Science and Technology, Sandec: department Sanitation, Water and Solid Waste for Development
Überlandstrasse 133, 8600 Dübendorf, Switzerland

*Corresponding author: linda.strande@eawag.ch

Table of contents

Supplementary information on data sources and statistical methods for data analysis
SI 1: Summary of references and inoculum properties of BMP tests used in this analysis... S2
SI 2: Summary of references and city, country location source of data ... S3
SI 3: Data preparation and statistical analysis ... S3
Supplementary figures and tables
SI 4: Spearman correlation matrix of a selection of physico-chemical parameters... S5
SI 5: Calculated free ammonia nitrogen (FAN)... S6
SI 6: Summary of results of Welch's ANOVA (TS)... S7
SI 7: Summary of results of Welch's ANOVA (pH)... S8
SI 8: Summary of results of Welch's ANOVA (NH_4^+) ... S9
SI 9: Silhouette plots and plots with first two principal components (Kampala, Uganda).... S10
SI 10: Technical and demographic survey information grouped (Kampala, Uganda)... S11
SI 11: Silhouette plots and plots with first two principal components (Lusaka, Zambia) ... S12
SI 12: Notched box plots illustrating three clusters (Lusaka, Zambia) ... S12
SI 13: Technical and demographic survey information grouped (Lusaka, Zambia)... S13
SI 14: Comparison of wastewater properties and survey data (Lusaka and Kampala)... S14
References... S15

Supplementary information on data sources and statistical methods for data analysis in this paper

We compiled results of BMP (biomethane potential) tests reported in the literature that included feces or excreta as substrate, and that followed the standard procedure for BMP tests,[1] or explained how modifications to the standard method were made. An overview of the study sources, substrates and inoculums is provided in Table SI 1.

SI 1: Summary of references and inoculum properties of BMP tests used in this analysis. AD: Anaerobic digestion. TS: Total solids. COD: Chemical oxygen demand.

Substrate	Inoculum	pH	TS (g/L)	COD (g/L)	NH_4^+ (mgN/L)	Free Ammonia Nitrogen (mgN/L)[b]	Incubation temperature (°C)	Source
Feces	Tank	7.89	29.0	35.4	896	27.5	20	Sam et al. (2022)[2]
Feces	Pit	7.67	39.8	86.1	3320	61.5	20	Sam et al. (2022)[2]
Feces	Pit	7.67	39.8	86.1	3320	174.5	37	Sam et al. (2022)[2]
Feces	AD sludge	7.52	21.9	18.4	938	34.9	37	Maqbool et al. (2024)[3]
Feces	AD sludge	-	-	-	-	-	35	Kim et al. (2019)[4]
Feces	AD sludge + saw dust	-	-	-	-	-	35	Riungu et al. (2019)[5]
Feces	Tap water[a]	-	-	-	-	-	25-35	Singh et al. (2021)[6]

[a]Mixed with tap water 1:1, [b]calculated at incubation temperature, without considering additional NH_4^+-input from the substrate.

We reported the conversion of substrate COD (chemical oxygen demand) to CH_4 as the measured maximum methane yield ($B_{o,meas}$) using equation Eq-1. For these calculations, we used a temperature of 20°C and the indicated CH_4 content in the biogas. If this value was not available, we assumed a 70% CH_4 content in the biogas, which has been previously reported as a maximum for anaerobic digestion of fresh feces and urine,[2] and for anaerobic digestion of fresh feces.[7]

$$B_o\_BMP = \frac{m_{BM} \times C_{CH4}}{COD_{in}} \qquad \textit{Eq-1}$$

Where:

$$Bo\_BMP = measured\ maximum\ methane\ yield, kg\ CH_4/kg\ COD$$
$$m_{BM} = Mass\ of\ biothemane\ produced\ from\ BMP\ test\ at\ 20°C\ [kg]$$
$$C_{CH4} = Mass\ of\ CH_4\ per\ Mass\ biomethane\ (assumed\ 0.7\ [kg/kg])$$
$$COD_{in} = Mass\ of\ COD\ in\ feces\ added\ to\ the\ BMP\ test\ [kg]$$

Although the values are from 9 different studies, they were collected and analyzed in a relatively comparative fashion as all conducted by the same research group

In all of the studies presented in Table SI 2**,** composite samples were collected from onsite containments with core sample or grab sample devices (combined bottom, middle and top), or after complete emptying of containments obtained from trucks at treatment plants (one beginning, two middle, and one end of discharge). In Lusaka, Zambia, all samples were collected in a fully randomized fashion across the entire city. In all other cities, samples were collected in a convenience-based fashion.

Samples were transported on ice to the laboratory, and analysis was conducted in the country where the sample was collected. In all studies, TS (total solids) and VS (volatile solids) were analyzed using the gravimetric method, and pH and electrical conductivity using a measurement probe. Ammonium (NH_4^+) was analyzed either using Hach Lange Test kits or the phenate method.[8] Survey data was with a questionnaire based on interviews and observations, to collect technical and demographic data. Further methodological information can be obtained in the publications, supplemental information and data repositories.

SI 2: Summary of references and location (city, country) of data for the 1,349 samples collected from onsite containments in 11 different countries used in this study.

Origin and date of sample collection	Link to data repository	Publication
Ougadougou, Burkina Faso<br>*Collected: December 2010 to February 2011, and July to August 2011*	-	Bassan et al. (2013)[9]
Dar es Salaam, Tanzania<br>*Collected: January to May 2017, and December 2017 to March 2018*	-	Esanju (2018), Marwa (2017)[10, 11]
Kampala, Uganda<br>*Collected: December 2013 to March 2014*	https://doi.org/10.25678/0000tt	Strande et al. (2018)[12]
Hanoi, Vietnam<br>*Collected: September 2013 to May 2014*	https://doi.org/10.25678/0000tt	Englund et al. (2020)[13]
Sircilla and Bengaluru, India<br>*Collected: August 2018 to March 2019*	https://doi.org/10.25678/0002VH	Prasad et al. (2021)[14]
Lusaka, Zambia<br>*Collected: September to December 2019*	https://doi.org/10.25678/00037X	Ward et al. (2021)[15]

Naivasha, Kenya<br>*Collected: March 2021*<br>Kampala, Uganda<br>*Collected: February 2021*	https://doi.org/10.25678/000702	Ward et al. (2023)[16]
Kumasi, Ghana;<br>Devanahalli, India;<br>Naivasha, Kenya;<br>Beirut and villages in the Bekaa Region, Lebanon;<br>Freetown, Sierra Leone; Kampala, Uganda;<br>Lusaka Zambia<br>*Collected: March 2021 to June 2022*	https://doi.org/10.25678/0007KN	Andriessen et al. (2023)[17]
Victoria, Canada<br>*Collected: April–June 2023*<br>Kampala, Uganda<br>*Collected: January–February 2024*	https://doi.org/10.25678/000FPN	Shaw et al. (2026)[18]

SI 3: Data preparation and statistical analysis to identify patterns between survey data and in situ properties

All data included in analysis were from stored wastewater ('fecal sludge') from onsite containments (e.g., pits, tanks). The data were composed of six physico-chemical parameters (i.e., TS, pH, NH_4^+, VS, COD and EC (electrical conductivity)), and nine related survey features (i.e., size of containment, lining of containment). The datasets were harmonized for nomenclature and units of physico-chemical parameters. Entries containing phrases such as "I don't know" were treated as missing values. Reported wastewater types were standardized to a controlled vocabulary. A binary variable (greywater added) was created to indicate whether any greywater source (kitchen, bathing, laundry, washing) was reported in addition to toilet inputs. Building types were reassigned to broader categories. Entries containing "household" were mapped to Household, while "commercial", "factory", "office", "mall", "restaurant", and "house of worship" were grouped under Commercial. Entries containing "public toilet" were mapped to Public toilet, and entries containing "school" to School. Containment types were harmonized by combining different expressions. Any entry containing "septic" was standardized to Tanks, while "pit latrine" or "soak pit" were grouped under Pits. Facilities described as "treatment" were set to missing values. A binary indicator (fully lined) was created based on no lining, partially lined (both "false") and fully lined ("true"). Reported toilet types were consolidated into a limited set of categories. "Cistern flush" entries were standardized as Cistern, while "pour flush" and "dry toilet" (dry toilets included all dry systems, including VIP and composting) were retained as separate

categories. Types associated with urine diversion, or unclassifiable pit latrines were removed (set to missing) due to inconsistent reporting. Household income data for Lusaka, Zambia were standardized into the four categories as very low (0–500 ZMK), low (501–1000 ZMK), middle (1001–2000 ZMK or "Medium"), and high (>2000 ZMK), whereas for Kampala it was reported in Strande et al. (2018)[12]. The number of reported users was recoded into a binary variable with two categories, as "less than 10" and "10 or more."

Statistical analysis was conducted using Python 3, next to standard libraries, scikit-learn (cluster, KMeans, PCA), scipy.stats (ttest-ind, chi2_contigency), statsmodels and pandas. Welch's ANOVA was employed to determine statistically significant differences among survey categories with respect to physico-chemical parameters. Silhouette plots and PCA were used to determine the number of clusters derived with the KMeans algorithm. For the ANOVA and the clustering, missing values were treated as NAN and not included in the analysis. Number of data points per combination for ANOVA are indicated in SI 6-SI 8.

Supplementary figures and tables referenced in the paper to provide additional or more in-depth information to the data analysis and results.

SI 4: Spearman correlation matrix of a selection of physico-chemical parameters of all data presented in Figure 2 with a complete data set of TS, pH, NH_4^+, VS COD and EC.

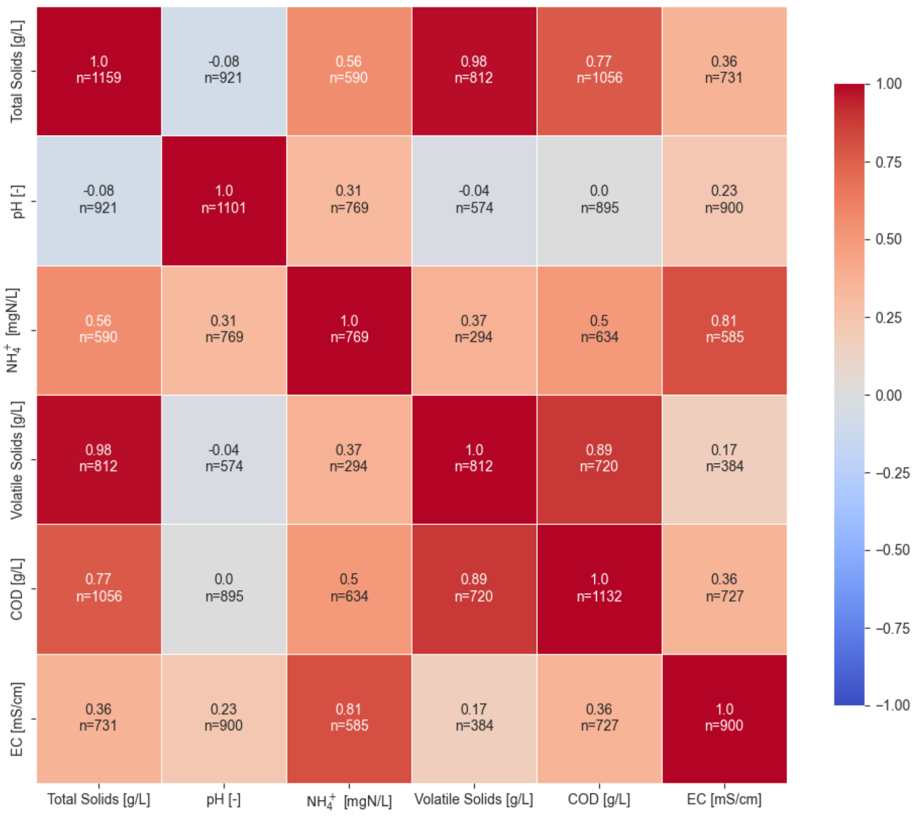

SI 5: Calculated free ammonia nitrogen (FAN) for the data used in this study separated by location. We calculated FAN using the *in situ* pH and NH_4^+, and an assumed temperature of 24°C.

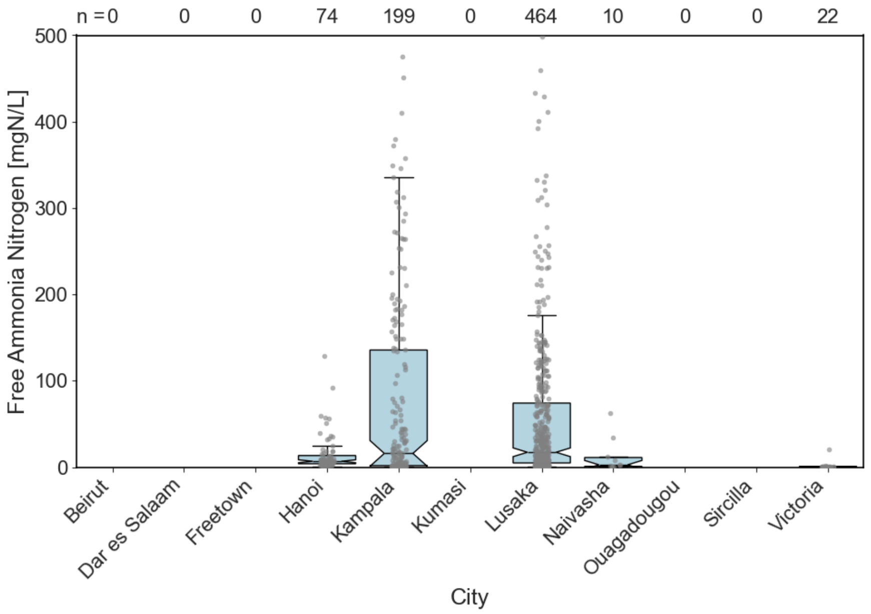

SI 6: Summary of results of Welch's ANOVA between technical and demographic survey data categories and TS. * for p-value < 5.00E-02, ** for p-value < 1.00E-02, *** for p-value < 1.00E-03.

TS	Overall			Kampala - all			Lusaka - all			Overall - Pits			Kampala - Pits			Lusaka - Pits			Overall - Tanks			Kampala - Tanks			Lusaka - Tanks		
**Category**	**P-value**		**N**	**P-value**		**N**	**P-value**		**N**	**P-value**		**N**	**P-value**		**N**	**P-value**		**N**	**P-value**		**N**	**P-value**		**N**	**P-value**		**N**
Containment	1.95E-15	***	1118	5.44E-03	**	207	8.23E-18	***	295																		
Fully lined	4.50E-02	*	318	7.31E-01		42	1.83E-02	*	133	6.05E-01		203	8.27E-01		6	1.64E-02	*	131	9.97E-02		76			31			
Greywater added	6.16E-03	**	1024	6.81E-03	**	206	3.34E-03	**	274	8.31E-01		458	2.47E-03	**	81	2.54E-03	**	122	8.14E-01		523	2.70E-01		120	5.91E-01		152
Income level	1.29E-01		1159	1.90E-03	**	207	5.52E-03	**	331	4.62E-03	**	519	3.69E-06	***	82	3.88E-01		141	2.75E-02	*	558	8.25E-01		120	9.84E-01		154
Building usage	2.85E-06	***	1064	4.10E-04	***	207	9.18E-07	***	296	4.68E-05	***	481	1.41E-02	*	82	4.34E-01		141	7.41E-06	***	539	5.49E-02		120	2.66E-03	**	153
Overflow	4.94E-11	***	487	7.04E-03	**	38	3.25E-06	***	285	1.01E-02	*	230			5	4.42E-02	*	137	6.11E-01		221	2.52E-02	*	30	6.29E-01		148
Flush type	1.34E-33	***	741	1.19E-01		42	2.80E-10	***	294	4.85E-12	***	345			6	8.98E-01		141	2.10E-04	***	354	6.37E-01		31	1.63E-01		153
Users >10	1.75E-02	*	1159	9.45E-02		207	3.04E-01		331	9.83E-03	**	519	4.77E-01		82	4.44E-01		141	8.89E-03	**	558	1.05E-01		120	1.88E-01		154
Water connection	2.96E-08	***	503	5.22E-01		42	1.85E-06	***	294	7.95E-01		236	2.18E-01		6	6.55E-01		141	4.63E-01		228	2.95E-01		31	7.62E-01		153

SI 7: Summary of results of Welch's ANOVA between technical and demographic survey data categories and pH. * for p-value < 5.00E-02, ** for p-value < 1.00E-02, *** for p-value < 1.00E-03.

**pH**	**Overall**			**Kampala - all**			**Lusaka - all**			**Overall - Pits**			**Kampala - Pits**			**Lusaka - Pits**			**Overall - Tanks**			**Kampala - Tanks**			**Lusaka - Tanks**		
**Category**	**P-value**		**N**	**P-value**		**N**	**P-value**		**N**	**P-value**		**N**	**P-value**		**N**	**P-value**		**N**	**P-value**		**N**	**P-value**		**N**	**P-value**		**N**
Containment	2.21E-07	***	1037	3.60E-10	***	218	2.68E-01		440																		
Fully lined	9.07E-01		413	2.43E-01		42	4.56E-01		228	2.77E-02	*	298	4.41E-01		6	3.03E-01		226	2.71E-02	*	76			31			
Greywater added	8.27E-05	***	1001	1.44E-07	***	217	6.29E-01		421	2.89E-01		474	9.81E-01		87	3.06E-01		217	7.39E-02		482	2.68E-01		125	9.83E-01		202
Income level	1.39E-18	***	1101	6.15E-12	***	218	1.70E-01		499	5.69E-10	***	497	2.22E-02	*	88	5.81E-01		236	1.40E-04	***	499	5.90E-02		125	3.35E-01		204
Building usage	1.88E-02	*	1040	2.78E-03	**	218	8.54E-01		442	7.98E-01		497	5.03E-01		88	9.44E-01		236	2.65E-02	*	498	2.52E-02	*	125	2.13E-02	*	203
Overflow	1.98E-02	*	633	6.73E-03	**	38	9.72E-01		431	4.32E-01		325			5	3.93E-01		232	5.03E-05	***	270	5.40E-02		30	1.30E-01		197
Flush type	1.35E-04	***	720	6.10E-03	**	42	1.78E-02	*	441	5.95E-01		355			6	6.63E-01		236	5.93E-09	***	321	6.78E-02		31	3.80E-02	*	203
Users >10	1.69E-03	**	1101	5.72E-01		218	4.63E-01		499	3.22E-04	***	497	6.35E-01		88	8.96E-01		236	7.57E-01		499	6.20E-01		125	2.56E-01		204
Water connection	1.28E-01		650	1.66E-04	***	42	4.33E-01		441	2.03E-01		331	6.67E-01		6	1.07E-01		236	6.49E-05	***	278	9.27E-03	**	31	7.02E-06	***	203

SI 8: Summary of results of Welch's ANOVA between technical and demographic survey data categories and NH_4^+. * for p-value < 5.00E-02, ** for p-value < 1.00E-02, *** for p-value < 1.00E-03.

NH_4^+	Overall			Kampala - all			Lusaka - all			Overall - Pits			Kampala - Pits			Lusaka - Pits			Overall - Tanks			Kampala - Tanks			Lusaka - Tanks		
**Category**	**P-value**		**N**	**P-value**		**N**	**P-value**		**N**	**P-value**		**N**	**P-value**		**N**	**P-value**		**N**	**P-value**		**N**	**P-value**		**N**	**P-value**		**N**
Containment	4.29E-71	***	711	1.04E-21	***	199	1.65E-64	***	408																		
Fully lined	1.07E-03	**	253			27	4.00E-01		196	2.52E-01		201			1	4.00E-01		196			50			26			
Greywater added	2.49E-12	***	692	1.90E-12	***	198	8.19E-11	***	402	1.54E-01		285	3.61E-01		80	5.46E-03	**	200	3.51E-02	*	403	3.06E-02	*	118	1.62E-01		200
Income level	2.30E-12	***	769	6.47E-15	***	199	2.11E-07	***	464	5.41E-03	**	292	3.94E-01		81	3.05E-01		206	1.66E-01		419	4.08E-03	**	118	4.03E-01		202
Building usage	8.16E-09	***	715	3.66E-04	***	199	2.42E-05	***	410	7.54E-03	**	292	4.65E-01		81	2.71E-01		206	3.60E-06	***	418	4.94E-04	***	118	2.00E-04	***	201
Overflow	5.77E-40	***	458	8.67E-01		27	3.63E-32	***	399	9.69E-02		208			1	1.60E-01		202	7.66E-02		246	8.49E-01		26	2.90E-01		195
Flush type	4.00E-42	***	468	1.30E-01		27	8.05E-35	***	409	8.41E-02		212			1	8.36E-01		206	5.08E-02		252	5.79E-02		26	3.68E-02	*	201
Users >10	7.34E-03	**	769	3.44E-02	*	199	5.61E-02		464	1.81E-01		292	8.78E-03	**	81	4.01E-01		206	3.59E-04	***	419	5.76E-01		118	5.07E-03	**	202
Water connection	1.29E-20	***	468	2.42E-01		27	1.31E-18	***	409	8.69E-01		212			1	9.48E-01		206	9.72E-02		252	1.22E-01		26	3.31E-01		201

SI 9: Silhouette plots and plots with the first two principal components and the defined clusters for the Kampala, Uganda data set. Silhouette plots were used to determine the number of clusters, by comparing the average silhouette scores (red dashed line). In this case, Clusters = 3 exhibited the highest silhouette score, which was used for further analysis. Plotting principal components was used to visually examine whether and how the clusters are separated from each other.

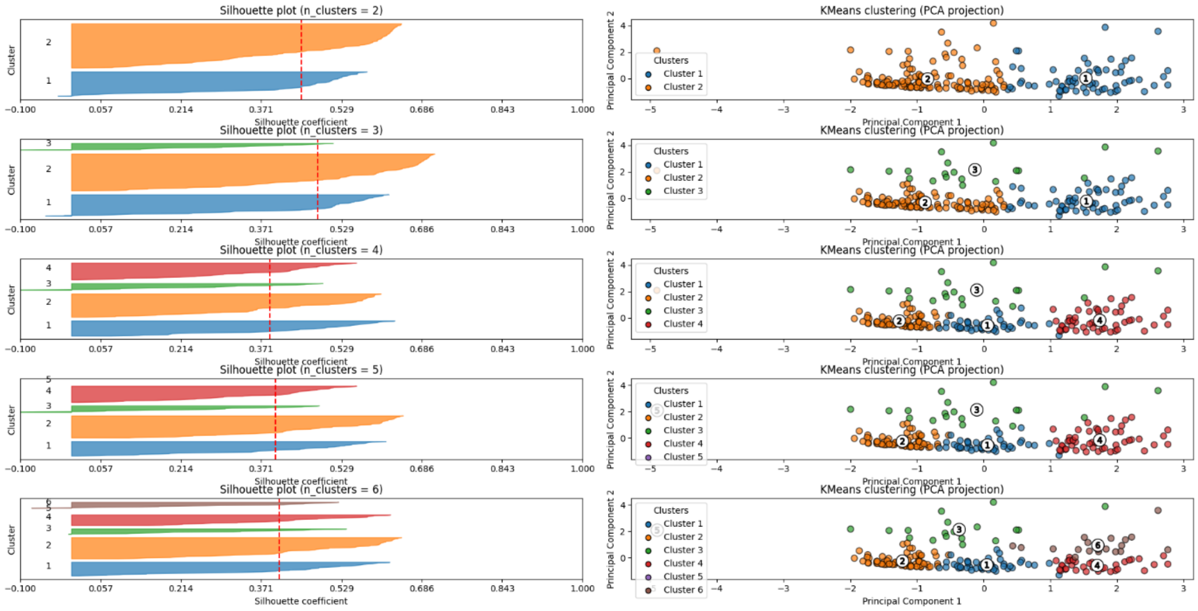

SI 10: Technical and demographic survey information grouped by clusters for Kampala, Uganda

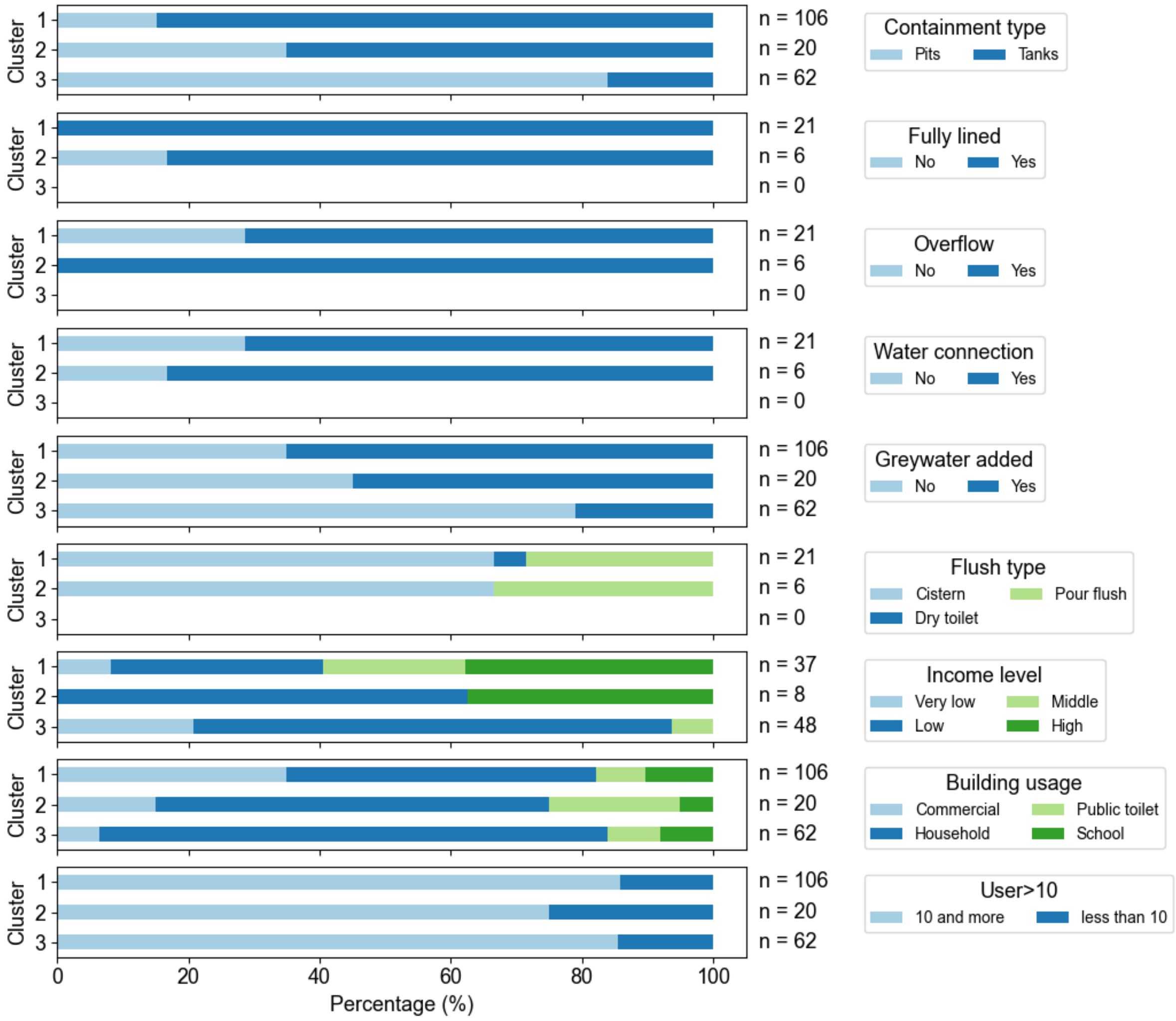

SI 11: Silhouette plots and plots with the first two principal components and the defined clusters for the Lusaka, Zambia data set. Silhouette plots were used to determine the number of clusters, by comparing the average silhouette scores (red dashed line). In this case, Clusters = 3 exhibited the highest silhouette score, which was used for further analysis. Plotting principal components was used to visually examine whether and how the clusters are separated from each other.

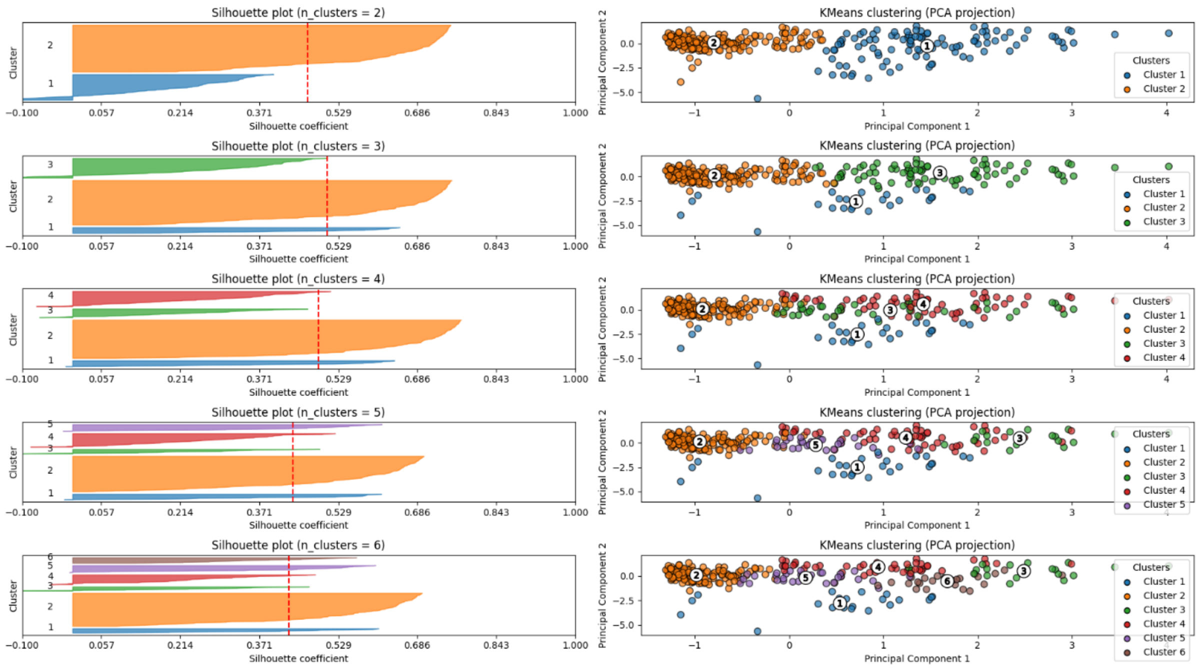

SI 12: Notched box plots illustrating three clusters of physico-chemical properties of wastewater collected in Lusaka, Zambia, based on TS, pH and NH_4^+. COD and calculated Free Ammonia Nitrogen included for illustration. No data available for Soluble COD / Total COD (E).

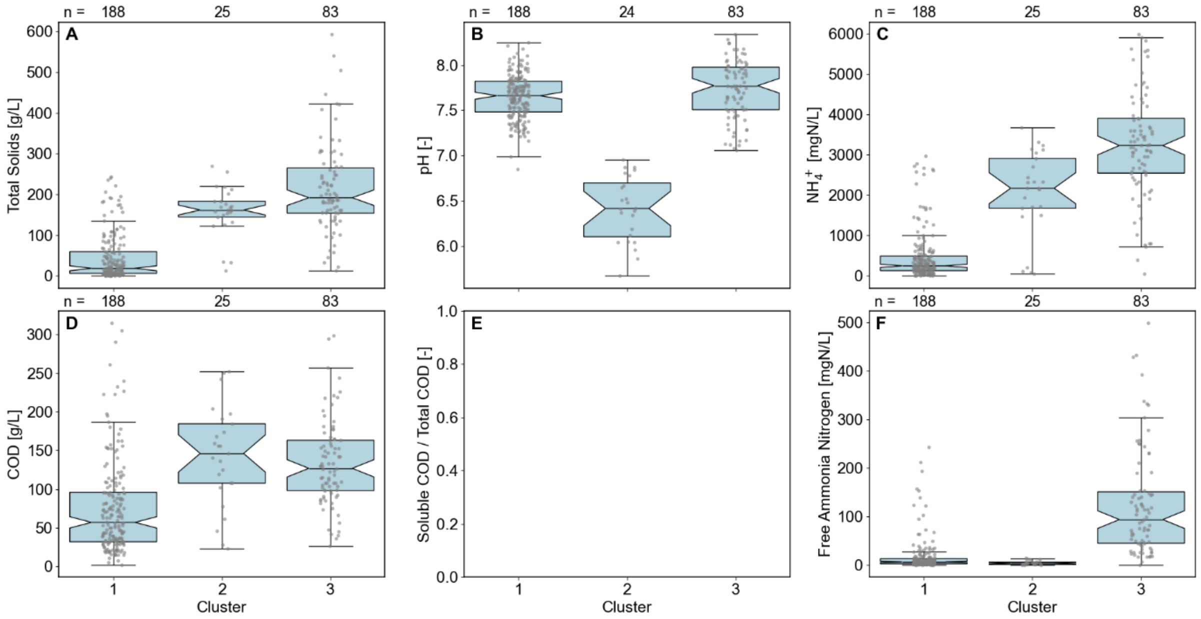

SI 13: Technical and demographic survey information grouped by clusters for Lusaka, Zambia.

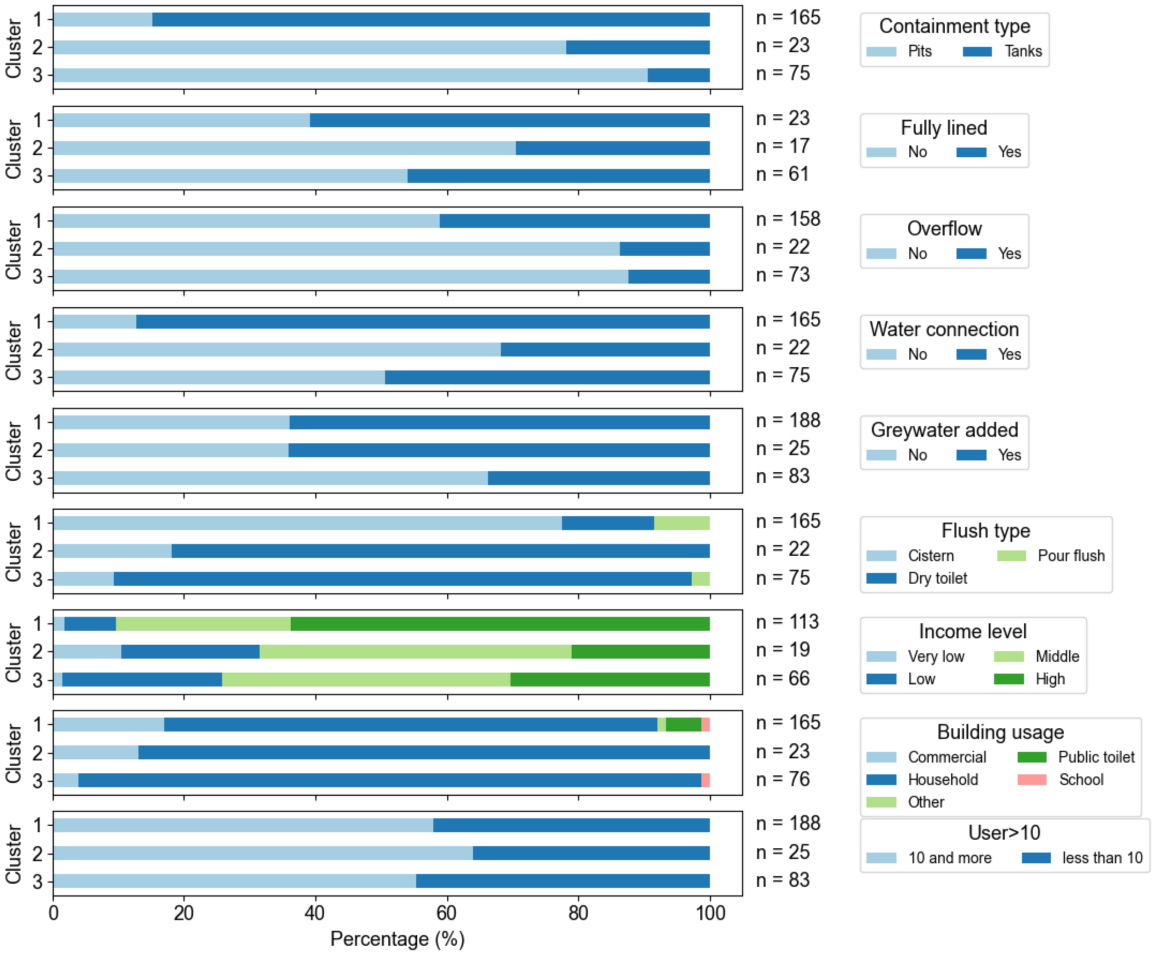

SI 14: Comparison of the wastewater properties and the survey data for Kampala and Zambia, separated by the clusters also presented in SI 9 to SI 13.

**Category**	Kampala **Cluster 1**	Zambia **Cluster 1**	Kampala **Cluster 2**	Zambia **Cluster 2**	Kampala **Cluster 3**	Zambia **Cluster 3**
Properties of wastewater						
TS (gTS/L)	6	19	64	162	24	192
pH (-)	7.4	7.7	7.1	6.4	8.4	7.8
NH_4^+ (mgN/L)	221	250	333	2’172	1’587	3’229
FAN (mgN/L)	2	6	2	3	185	93
Technical data containment by %						
**Containment**						
Pits	15	15	35	78	84	91
Tanks	85	85	65	22	16	9
**Fully lined**						
No	0	39	17	71	0	54
Yes	100	61	83	29	0	46
**Overflow**						
No	29	59	0	86	0	88
Yes	71	41	100	14	0	12
Technical data water usage by %						
**Water connection**						
No	29	13	17	68	0	51
Yes	71	87	83	32	0	49
**Greywater added**						
No	35	36	45	36	79	66
Yes	65	64	55	64	21	34
**Flush type**						
Cistern	67	78	67	18	0	9
Dry	5	14	0	82	0	88
Pour	29	8	33	0	0	3
Demographic data by %						
**Building usage**						
Household	47	75	60	87	77	95
Very low	*8*	*2*	*0*	*11*	*21*	*2*
Low	*32*	*8*	*63*	*21*	*73*	*24*
Middle	*22*	*27*	*0*	*47*	*6*	*44*
High	*38*	*64*	*38*	*21*	*0*	*30*
Non-household						
Public toilets	8	5	20	0	8	0
School	10	1	5	0	8	1
Commercial	35	18	15	13	6	4
**Users**						
>10	86	58	75	64	85	55
<10	14	42	25	36	15	45

References

(1) Holliger, C.; Alves, M.; Andrade, D.; Angelidaki, I.; Astals, S.; Baier, U.; Bougrier, C.; Buffière, P.; Carballa, M.; de Wilde, V.; et al. Towards a standardization of biomethane potential tests. *Water Science and Technology* **2016**, *74* (11), 2515–2522. DOI: 10.2166/wst.2016.336 (accessed 10/23/2025).
(2) Sam, S. B.; Ward, B. J.; Niederdorfer, R.; Morgenroth, E.; Strande, L. Elucidating the role of extracellular polymeric substances (EPS) in dewaterability of fecal sludge from onsite sanitation systems, and changes during anaerobic storage. *Water Research* **2022**, *222*, 118915. DOI: 10.1016/j.watres.2022.118915.
(3) Maqbool, N.; Sam, S.; Jamal Khan, S.; Strande, L. Relation of organic fractions in fresh and stored fecal sludge and foodwaste to biogas production. *Journal of Water, Sanitation and Hygiene for Development* **2024**, *14* (3), 277–290. DOI: 10.2166/washdev.2024.319.
(4) Kim, J.; Kim, J.; Lee, C. Anaerobic co-digestion of food waste, human feces, and toilet paper: Methane potential and synergistic effect. *Fuel* **2019**, *248*, 189–195. DOI: https://doi.org/10.1016/j.fuel.2019.03.081.
(5) Riungu, J.; Ronteltap, M.; van Lier, J. B. Anaerobic stabilisation of urine diverting dehydrating toilet faeces (UDDT-F) in urban poor settlements: biochemical energy recovery. *Journal of Water, Sanitation and Hygiene for Development* **2019**, *9* (2), 289–299. DOI: 10.2166/washdev.2019.099 (accessed 3/10/2026).
(6) Singh, S.; Hariteja, N.; Sharma, S.; Raju, N. J.; Prasad, T. J. R. Production of biogas from human faeces mixed with the co-substrate poultry litter & cow dung. *Environmental Technology & Innovation* **2021**, *23*, 101551. DOI: 10.1016/j.eti.2021.101551.
(7) Poocheera, S.; Suntivarakorn, R.; Treedet, W. Biogas Production from Human Faece and Community Waste Food. *Advanced Materials Research* **2014**, *931-932*, 1101–1105. DOI: 10.4028/www.scientific.net/AMR.931-932.1101.
(8) Apha. *Standard methods for the examination of water and wastewater*; Apha, 1985.
(9) Bassan, M.; Tchonda, T.; Yiougo, L.; Zoellig, H.; Mahamane, I.; Mbéguéré, M.; Strande, L. Characterization of faecal sludge during dry and rainy seasons in Ouagadougou, Burkina Faso. **2013**.
(10) Esanju, M. M. Assessment of the applicability of data collection methodology for estimating quantities and qualities of faecal sludge Q&Q model in Dar es Salaam. Master's thesis, University of Dar es Salaam, Dar es Salaam, 2018. https://lib.udsm.ac.tz/bib/139472.
(11) Marwa, L. I. Development of potential predictors for faecal sludge characterisation on a community scale, the case study of Mlalakua Community, Dar es Salaam. Master's thesis, University of Dar es Salaam, Dar es Salaam, 2017.
(12) Strande, L.; Schoebitz, L.; Bischoff, F.; Ddiba, D.; Okello, F.; Englund, M.; Ward, B. J.; Niwagaba, C. B. Methods to reliably estimate faecal sludge quantities and qualities for the design of treatment technologies and management solutions. *Journal of environmental management* **2018**, *223*, 898–907.
(13) Englund, M.; Carbajal, J. P.; Ferré, A.; Bassan, M.; Hoai Vu, A. T.; Nguyen, V.-A.; Strande, L. Modelling quantities and qualities (Q&Q) of faecal sludge in Hanoi, Vietnam and Kampala, Uganda for improved management solutions. *Journal of Environmental Management* **2020**, *261*, 110202. DOI: 10.1016/j.jenvman.2020.110202.
(14) Prasad, P.; Andriessen, N.; Moorthy, A.; Das, A.; Coppens, K.; Pradeep, R.; Strande, L. Methods for estimating quantities and qualities (Q&Q) of faecal sludge: field evaluation in Sircilla, India. *Journal of Water, Sanitation and Hygiene for Development* **2021**, *11* (3), 494–504. DOI: 10.2166/washdev.2021.269 (accessed 5/5/2025).
(15) Ward, B. J.; Andriessen, N.; Tembo, J. M.; Kabika, J.; Grau, M.; Scheidegger, A.; Morgenroth, E.; Strande, L. Predictive models using “cheap and easy” field measurements: Can they fill a gap in planning, monitoring, and implementing fecal sludge management solutions? *Water Research* **2021**, *196*, 116997.
(16) Ward, B. J.; Nguyen, M. T.; Sam, S. B.; Korir, N.; Niwagaba, C. B.; Morgenroth, E.; Strande, L. Particle size as a driver of dewatering performance and its relationship to stabilization in fecal sludge. *Journal of Environmental Management* **2023**, *326*, 116801. DOI: 10.1016/j.jenvman.2022.116801.

(17) Andriessen, N.; Appiah-Effah, E.; Browne, S. J. I.; al Jahjah, R.; Kabika, J.; Kinobe, J. R.; Korir, N.; Nishimwe, P.; Niwagaba, C. B.; Pradeep, R.; et al. Quantities and qualities of fecal sludge: Experiences from field implementation with a Volaser in 7 countries during a pandemic. *Frontiers in Water* **2023**, *Volume 5 - 2023*, Original Research. DOI: 10.3389/frwa.2023.1130081.
(18) Shaw, K.; Niwagaba, C. B.; Strande, L.; Dorea, C. C. Improving Methane Emissions Estimates from Non-Sewered Sanitation Containments for Global Application. *Environmental Science & Technology* **2026**, *60* (20), 14467–14481. DOI: doi.org/es-2025-17325w.
